# Hierarchy and diffusion of organizational forms

**DOI:** 10.3389/fpsyg.2022.932273

**Published:** 2022-07-29

**Authors:** Guido Fioretti, Martin Neumann

**Affiliations:** ^1^Department of Management, University of Bologna, Bologna, Italy; ^2^Institute of Sociology, Johannes Gutenberg University Mainz, Mainz, Germany

**Keywords:** evolutionary social science, organizational ecology, organizational routines, organizational forms, information flows, T-pattern

## Abstract

In this paper we first of all summarize and rationalize current typologies of organizational forms, arranging available classifications in a hierarchy of increasing generality. The ensuing structure parallels the classification of living beings into classes of increasing generality such as species, genus, family, order, and so on. Subsequently, we analyze the structure of communications that favored the diffusion of each organizational form. We isolate a few stylized communication structures, pointing to the presence of several sources endowed with global connections as the most efficient diffusion mode. The empirical research that is being carried out on single organizations is close to observing their T-patterns, whereas nothing comparable is in sight for organizational forms as yet. However, at least in some cases, we dare to formulate tentative hypotheses on certain features that the ensuing T-patterns-of-patterns might exhibit.

## Introduction

There exists an evolutionary interpretation of human societies which likens organizations to organisms, and their norms, routines, and culture to their genome. This analogy is based on the observation that organizations receive a certain imprinting at foundation, which they do not change throughout their existence (Nelson and Winter, 1982; Hannan and Freeman, 1989). For instance, *Apple* was founded with the aim of producing desktops that would excel in user-friendliness and graphics while addressing the upper end of the market. Today, after switching to portable devices, this focus stayed. Likewise, one may observe that organized religions fix most of their dogmas in the early centuries of their existence, that most States write a Constitution while they are being founded, and so on.

The idea is that the norms, culture, and routines that are set at foundation and during infancy provide an imprinting that single organizations cannot change afterward, albeit novel organizations can be created with different cultures, norms, and routines. Thus, the population of organizations does change, whereas single organizations largely do not.

Obviously, this claim should not be taken too literally. Nobody denies that organizational change takes place, for organizations must continuously adapt to a mutable and unpredictable environment in the course of their existence. What this approach suggests is that organizational change can only take place insofar it does not destroy an organization’s identity, which in its turn can only change at a much slower time scale than the organization itself (Levinthal, 2021). Such qualifications do not destroy the analogy with living organisms, which also adapt their genome to changing circumstances by means of epigenetic mechanisms (Bird, 2007).

One implication of this conceptual scheme is that just like living organisms belong to species, human organizations pertain to a limited number of organizational forms that can be used as templates any time a new organization is founded. In this article, we submit that, just like living organisms can be arranged in classes of increasing generality such as species, genus, family, and other groupings, also human organizations can be arranged in a hierarchy of organizational forms of increasing generality depending on the aspect one looks at. Moreover, we submit that the current inability of evolutionary social science to define what constitutes an organizational form (Romanelli, 1991) originates—among else—from focusing on different aspects of organizations, which in our scheme pertain to different generality levels. Thus, with our hierarchy of organizational forms, we hope to contribute a much-needed conceptual clarification.

Henceforth, we will show that this hierarchy helps make sense of changing information structures within organizations and organizational forms, as well as their origin and diffusion in society at large. In particular, we shall highlight that information about organizational forms diffused in society along different paths depending on the epoch of their invention and the actors who conceived them. By providing a comprehensive framework for information structures at different aggregation levels of human organizations, we hope to offer a conceptual contribution to the comparison of information structures at the human and the nanoscale (Magnusson, 2020). In particular, we highlight that in several instances, there existed several centers from which information radiated, a circumstance that is likely to have generated faster dynamics than either pure broadcast from one single source, or local diffusion.

Our contribution is admittedly more limited insofar as it concerns T-patterns (Magnusson, 2020), for the empirical research on organizational routines is still extremely limited. Only a few organizational routines have been recorded, though it is interesting to remark that patterns have been found, indeed (Hutchins, 1991; Egidi and Narduzzo, 1997; Pentland et al., 2010, 2011). Conceivably, if such analyses would be carried out on large numbers of organizations, the typical time patterns that characterize specific forms would be identified. The current state of research is very far from such a minimal goal, but we point to software engineering as a field where qualitative codification of behavior patterns has made substantial progress (Coplien and Harrison, 2005).

Empirical research is even farther removed from the ability to detect the patterns-of-patterns that likely characterize organizational forms of increasingly higher generality. However, we shall formulate hypotheses concerning possible features of these higher-order patterns.

The rest of this article is organized as follows. The next section illustrates our hierarchy of organizational forms ordered by increasing generality. In this section, we also formulate our hypotheses regarding a few likely features of their T-patterns. Subsequently, we discuss the historical record of their diffusion highlighting the structural properties of the information channels that were used as well as, whenever available, the degree of information centralization within each organizational form. Finally, we discuss a taxonomy of diffusion processes based on communication structure.

## Organizational forms

Life scientists identify species out of features that can be observed unambiguously, such as lack of inter-breeding or other indisputable evidence. This is hardly the case for social scientists, who are compelled to make subjective judgments whenever they define an organizational form.

The objective difficulty to ground organizational features on uniquely identifiable indicators has led to endless discussions as to what exactly constitutes an organizational form, how it can be identified, and where are its boundaries, without ever reaching unanimous conclusions (McKelvey, 1982; Hannan and Freeman, 1986; Romanelli, 1991; Pólos et al., 2002; Hannan et al., 2007). In the end, a sort of case-to-case pragmatism has prevailed where organizational forms are defined on indicators that suit each specific investigation, accepting subjectivity as an unavoidable feature of social research (Hannan and Freeman, 1989; Bogaert et al., 2016).

The vast majority of empirical research on organizational forms focuses on combinations of behavioral and technological features, e.g., the emergence of microbreweries as an organizational form distinct from large-scale industrial breweries (Carroll and Swaminathan, 1992, 2000), or multiteam systems whose communication is eventually mediated by information technologies (Mathieu et al., 2002; Zaccaro et al., 2020). This combination of technology and behavior to some extent resembles socio-technical systems research (Trist, 1981), but adds to it an emphasis on typical behavioral patterns that classical socio-technical investigations did not have.

A second stream of research defines organizational forms with respect to structure, originally contrasting multidivisional structures, also called M-form, to more traditional functional structures (Chandler, 1962), but later on extending the analysis to more nuanced structural features such as franchising (Brickley and Dark, 1987; Michael, 2000) or ownership structure (Hasan and Lozano-Vivas, 2002; Erhemjamts and Leverty, 2010). We submit that whenever organizational forms are defined by structure rather than behavior patterns, they simply pertain to a greater level of generality in the sense that structure may constrain, but does not precisely determine behavior. In our hierarchy of organizational forms, just like an organism belongs to a species as well as a genus, an organization can be an instance of an organizational form defined by its typical behavior patterns as well as a form defined by its structure.

Since nested classifications can be useful in many respects, we propose to define organizational forms on two other dimensions whereby organizations are eventually classified, the institutional dimension—such as being a bureaucracy—and the dimension of organizational ideology. While understanding institutional arrangements as organizational forms is rare but in use (Zucker, 1983; Lee and Pennings, 2002; Hallett and Ventresca, 2006), we found only one instance of an organizational form having been defined on ideology (Schneiberg et al., 2008). Albeit both usages are admittedly uncommon, we submit that substantial advantages can be obtained by adding these dimensions to our understanding of organizational forms. These additional two levels complete a nested scale of four levels, all of which enable us to define organizational forms that exhibit distinct patterns of diffusion along specific information channels and are likely to exhibit their own distinct T-patterns.

**Figure 1:**
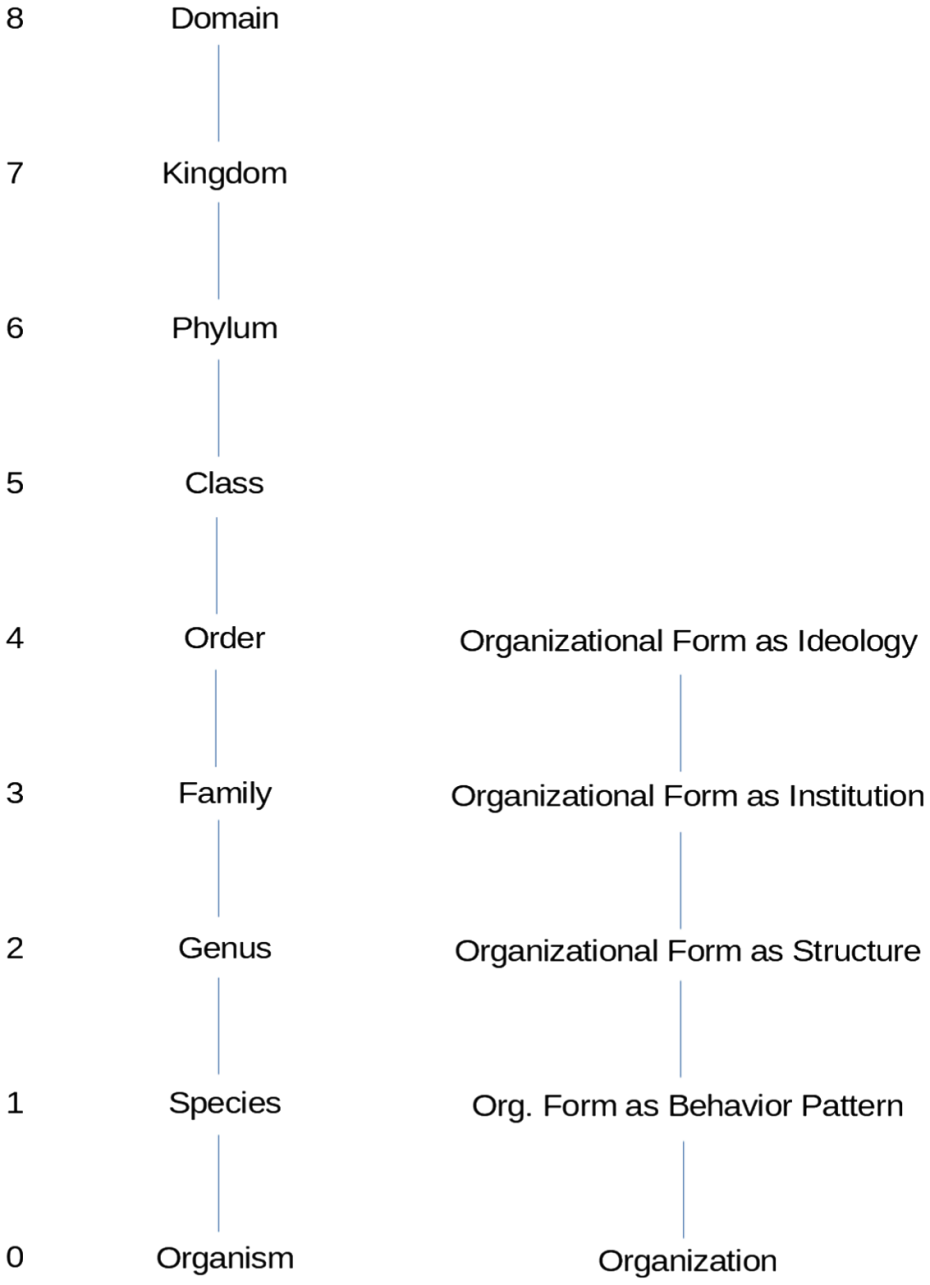
Hierarchical classification of living organisms (left) and human organizations (right). The accepted classification of living organisms entails eight levels nested in one another, e.g., Cleopatra is a cat but also a feline and a mammal. For human organizations, we propose four nested levels of organizational forms based on existing classification criteria. Each level is nested in the superior one; hence, each organization displays behavior patterns of a certain sort, has a typical structure, a certain institutional arrangement, and the organization is characterized by a certain ideology.

Figure 1 illustrates our nested classification of organizational forms aside from the corresponding classifications of living organisms. At the lowest level, we identify organizational forms defined on behavioral patterns (Level 1), which are included in organizational forms defined by structure (Level 2), institutional arrangement (Level 3), and ideology (Level 4). Since each subsequent level includes the previous one in a scale of increasing generality, one specific organization pertains to a form defined by its typical behavior patterns, as well as a form defined by its typical structure, a form defined by a specific institutional arrangement, and a form defined by its ideology.

One important difficulty experienced by the evolutionary understanding of organizations is that, contrary to living species, organizational hybrids do exist. Organizational forms are still useful as pure ideal types to which real organizations can be compared (Weber, 1922), but the distance between theory and the real world is larger than in the life sciences. In this respect, our nested scheme helps reducing this distance because organizations that would have appeared as hybrids along the vertical axis simply belong to organizational forms defined at different generality levels. By contrast, hybrids along the horizontal axis still blur the picture.

Henceforth, we shall describe a few organizational forms that have been identified at each of the above levels, positing for each of them the sort of T-patterns that might be observed. We shall start with behavior patterns to proceed with structure and institutional arrangement and conclude with ideology.

### Patterns of behavior

The simplest class of organizational forms is based on technological and behavioral features, entailing items such as micro-breweries (Carroll and Swaminathan, 1992, 2000) or multiteam systems making use of specific communication routines and technologies (Mathieu et al., 2002; Zaccaro et al., 2020). Unfortunately, in spite of a substantial number of empirical investigations, scholars could not agree on what exactly constitutes one such organizational form (Romanelli, 1991). Tentative definitions have mostly pointed to processes generative of organization boundaries (Hannan and Freeman, 1986), or behavioral codes that generate organizational identities (Pólos et al., 2002).

One attempt at uniting these insights, which we wholeheartedly join, has been recently proposed by Fiol and Romanelli (2012). Upon remarking that stable patterns of behavior are a universal feature of human organizations, they focused on the communities of practice where these patterns arise and are eventually adopted. In a nutshell, such behavior patterns may become sufficiently stable to be perceived as organizational behavior codes, which in their turn contribute to organizational identity while erecting boundaries that separate organizations from one another. One remarkable feature of this approach is that it justifies the usage of T-patterns in order to identify organizational forms.

**Figure 2:**
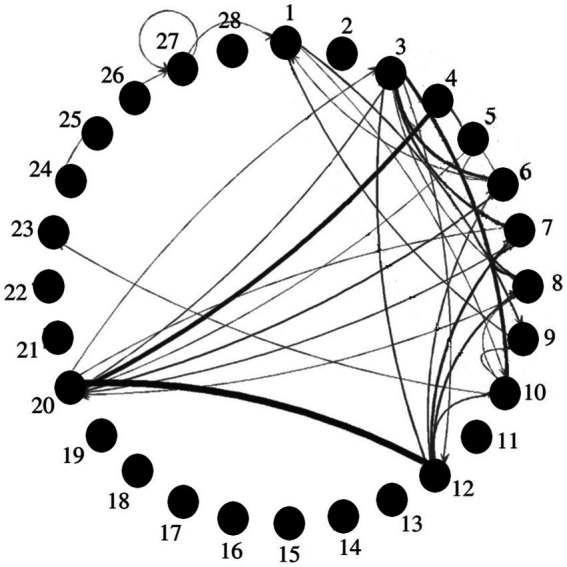
The graphical representation of behavioral routines that emerges out of empirical investigations. Nodes represent actions, edges link actions that are carried out in sequence, thickness represents their frequency. It is evident that routines do not merely repeat sequences of actions, but also add some degree of exploration to the received sequence. Our elaboration, loosely inspired by Pentland et al. (2011).

Research on organizational routines has focused on patterns that eventually emerge in the sequence of operations that organization members carry out (Hutchins, 1991; Egidi and Narduzzo, 1997; Pentland et al., 2010, 2011). Figure 2 illustrates the sort of data produced by these investigations. The nodes represent actions that could be carried out, edges connect actions that were carried out in sequence.

Such detailed empirical investigations on organizational routines are still rare, but they may become more common in the future. Thus, at least in principle organizational forms based on behavioral patterns could be detected by identifying their typical routines.

For instance, Pentland et al. (2010, 2011) analyzed the routines recorded by one and the same software in as diverse organizations as a labor union, a statistical research institute, a construction company, and a meat packaging plant. They purposedly selected organizations that were as different as possible from one another in order to extract different routines, but if data had been collected for—just to make an example—a set of micro-breweries and a set of large, mass-production breweries, we may expect that the T-patterns of the microbreweries would share common features, as well as those of the large breweries. To the extent that the common features of the firms in the first group would be different from the common features of the firms in the second group, these differences could have been used to discriminate them as belonging to two distinct organizational forms.

A remarkable step in this direction has been made in the field of software engineering, where behavioral patterns have been qualitatively identified (Coplien and Harrison, 2005). If these qualitative patterns could be expressed as T-patterns, then we would have a quantitative definition of an organizational form based on the coding patterns of software houses.

Notably, in the above example, one single software house would exhibit T-patterns, whereas the organizational form “software houses” (Level 1) would actually be defined on patterns-of-patterns. Thus, henceforth higher-order patterns will be denoted as T-patterns*^n^*, where *n* is the generality level of organizational forms in our scale. For instance, one single software house would generate T-patterns, whereas all software houses would be expected to exhibit T-patterns^1^.

### Structure

Contrary to organizational forms based on behavior patterns, those based on structural features were first introduced without any reference to evolutionary theory (Chandler, 1962). Nevertheless, they perfectly fit into our scheme as a higher-level classification that can include forms defined on behavior patterns. The structure generally changes at a slower time scale than patterns of behavior; hence, it makes sense to understand organizational dynamics as being framed by a structure that stays for some time, channels information flows and thereby enables and constrains patterns of behavior without specifying their details.

Several structure-based, Level 2 organizational forms have been identified in the literature. For any organization larger than a handful of individuals, the *functional structure* is the most obvious and the most common among organizations of any sort. It simply consists of arranging in separate units all those who carry out similar activities. In businesses, typical functions are Procurement, Production, and Sales, as well as Marketing or Research & Development.

Some business functions are linked by precedence relations, e.g., procurement comes first, then production, then sales. It is known that this is sufficient to generate oscillations of production (Sterman, 1989). We would not claim that T-patterns^2^ of functional structures are necessarily oscillatory, but they could exhibit oscillations even in absence of exogenous disturbances.

A more complex structure is generally adopted by organizations that carry out highly differentiated activities, such as large corporations that are active in different markets, or different geographical areas. Since organizations of this sort must adapt to the different environments where they operate, they create semi-autonomous divisions wherein functions are duplicated. The *multidivisional structure*, also called the M-form (Chandler, 1962), is another structure-based organizational form defined at Level 2. If and when its T-patterns^2^ will be observed, we hypothesize that they will differ markedly across its component divisions.

Still at Level 2, the *matrix structure* has two or more bundles of authority lines, for instance, one along functions (as in a functional structure) and the other one along markets or projects. Matrix structures contradict the principle of unity of command and the very fact that their members have two or more bosses makes for difficult decision-making. However, precisely, this feature enables it to combine expertise from different areas in the organization (Galbraith, 2009). It seems sensible to hypothesize that the more diverse fields are combined and the less repetitive its activities, the less regular T-patterns^2^ will be observed.

**Figure 3:**
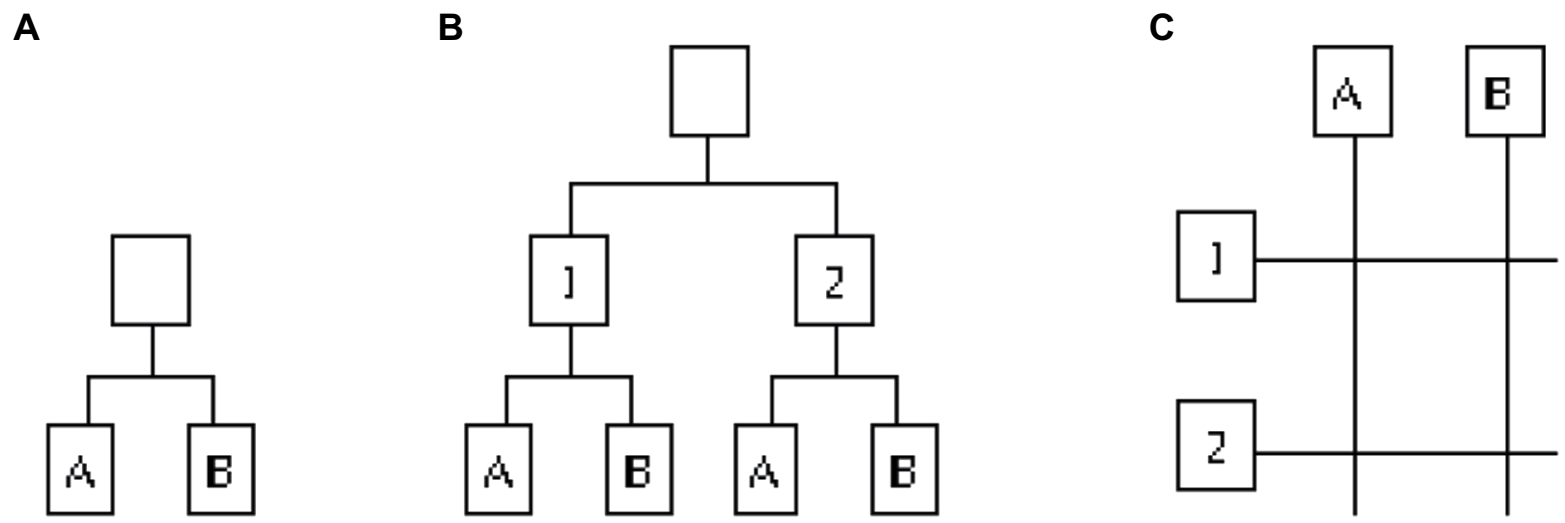
Three basic structures, typical of organizational forms at Level 2. Left **(A)**, a functional structure with two functions A and B. Center **(B)**, a multidivisional structure where functions A and B are duplicated across divisions 1 and 2, respectively. Right **(C)**, a matrix structure whose members report to functional units A and B as well as market/area/project specialists 1 and 2, respectively.

Figure 3 illustrates, left to right, a functional structure, a multidivisional structure, and a matrix structure, respectively. While functional and multidivisional structures are invariant with time, there exist versions of the matrix structure—also called *project organizations*—where the projects heading the horizontal lines change with time, as it happens for instance in large engineering firms. In these cases, a time sequence of structures would be more appropriate.

Finally, *adhocracies* (Mintzberg, 1979), also called *network organizations* (Miles and Snow, 1986), achieve maximum flexibility by quickly adapting their structure to changing environmental conditions. Adhocracies spearhead a trend toward increasingly flexible organizations, whose origin may be placed with the sociotechnical systems of the 1950s (Trist et al., 1963) but which greatly accelerated with lean manufacturing in the 1980s (Sugimori et al., 1977) and recently reached a remarkable peak with flat organizations composed uniquely by work teams indirectly coordinated by one chief executive officer (CEO), and no middle management in between (Bernstein et al., 2016). However, even in these extreme cases, organizational flexibility is limited by clear boundaries to the activities that organization members can undertake (Cabri and Fioretti, 2022). These organizations are capable of extreme plasticity, but only within a given set of possible structures (Levinthal and Marino, 2015). To an even greater extent than in the case of matrix structures, we hypothesize that in adhocracies T-patterns^2^ will only appear if flexibility is kept at quite a low level.

There exists a debate whether adhocracies/network organizations constitute a novel organizational form, or are rather just a variation around the theme of bureaucracy (Hales, 2002). Since we conceive organizational forms as nested in one another at increasing levels of generality, we can answer positively to both ends of this conundrum. In our interpretation, adhocracies/network organizations constitute a novel organizational form at the structural level (Level 2), while at the same time, they are still a bureaucracy at the level of the organizational forms defined as institutional arrangements (Level 3). Furthermore, our scheme can accommodate the observation that adhocracies often have a high ideological content (Mintzberg, 1979) by including them in an organizational form defined by ideology (Level 4).

### Institutional arrangements

Social scientists understand institutions as either rules, norms, conventions, habits that regulate organizational behavior, or specific organizations that issue those rules (Khalil, 1995; Edquist and Johnson, 2005; Greenwood et al., 2014). Henceforth, we shall adopt the first meaning, eventually employing the expression “institutional arrangement” in order to stress our choice.

One prominent institutional arrangement is the modern bureaucracy, often regarded as paradigmatic of capitalism itself (Weber, 1922). Occasionally, the norms of bureaucracy are used to call it an organizational form (Zucker, 1983; Lee and Pennings, 2002; Hallett and Ventresca, 2006). Albeit this understanding of bureaucracy is uncommon, we judge that it fits our scheme perfectly well.

Thus, we introduce a Level 3 where organizational forms are defined as institutional arrangements, more general than the Level 2 where organizational forms are defined by structure, which in its turn is more general than the Level 1 where organizational forms are defined by their behavior patterns. To our knowledge, three broad organizational forms have been identified at this Level 3.

We already hinted at bureaucracy as the first instance. Weber (1922) is credited for having highlighted the specific features of modern bureaucracies, above all their ability to define roles and positions before someone has been found to fill them. Weber’s bureaucracies, also called *machine bureaucracies* within organization studies (Mintzberg, 1979), are typical of corporations as well as public administrations and probably comprise the vast majority of human organizations today. A clear set of norms, generally made explicit by laws and internal regulations, specify the rights and duties of their members with respect to the organization as well as the relationships with colleagues. These norms result from the huge efforts these organizations typically make to standardize and regulate their operations in as much detail as possible, resulting in somewhat impersonal, but extremely efficient collective machines. Albeit in common parlance bureaucracies are negatively stymied because of their rigidity, organization theorists generally stress that no other organizational form can mobilize collective energies and pursue complex long-term goals to any comparable extent. Machine bureaucracies extend individual means-ends calculative rationality to collective action, eliminating arbitrariness and personal evaluation to an extent unrivaled by any other organizational form (Kalberg, 1980).

Mintzberg (1979) observed that there exists a variety he called *professional bureaucracy*, which includes hospitals and universities among its most notable instances, where standardization is not carried out within each single organization but rather by some external body representing professional interests, e.g., congresses and specialized journals for physicians and scientists. New theories and new clinical practices must obtain legitimation worldwide, a circumstance that typically makes innovation more difficult for professional bureaucracies in comparison to machine bureaucracies. However, professional bureaucracies generally concede their members substantially higher margins of discretion in comparison to machine bureaucracies.

The third category entails organizations where standardization is minimal or absent, bonds between members being largely based on personal relations where favors are made in exchange for unconditional dependence. The members of *patronage-based* organizations (Flap, 1990) are coopted into unequal relations characterized by unlimited loyalty and are expected to follow their patron in their fortunes and misfortunes (Redding, 1985). For instance, new CEOs typically replace the whole top management as soon as they are nominated (Jackall, 1988), but also political parties require fidelity from elected parliament members, whereas criminal gangs are possibly the most extreme instance. Small organizations may slip into patronage when personal relations are more important than rules, but they are also capable of more balanced, sometimes even egalitarian arrangements.

We hypothesize that homogeneous T-patterns^3^ will be most easily observed among machine bureaucracies, least easily observed among patronage-based organizations, with professional bureaucracies somewhere in between. Furthermore, we hypothesize that machine bureaucracies will likely exhibit homogeneous T-patterns^3^ even when they grow large, whereas this may not be the case for large professional bureaucracies (where, for instance, university professors may hold “chairs” that are substantially independent of one another) as well as some large patronage-based organizations (for instance, political systems characterized by a large number of heterogeneous parties and cleavages within each of them).

### Ideology

Ideologies provide a neat interpretation of social phenomena, calling for personal commitment to realize a desired change that is often conceived as a struggle against some figurative or very concrete enemy (Cranston, 1979). Religions are generally not equated to ideologies because of their supernatural aims, but they may acquire an ideological character insofar as their ethos is directed toward mundane objectives (Williams, 1996).

Ideologies concur to build organizational identity by providing a common purpose to their members (Alvesson, 1987) and easing organizational decision/making (Brunsson, 1982). Distinct organizations that share a specific ideology are eventually grouped together and contrasted to competing groups, for instance, in the case of cooperative enterprises (Simons and Ingram, 2004; Schneiberg et al., 2008) or, more recently, corporations leaning toward opposite political ideologies (Gupta and Briscoe, 2020; Swigart et al., 2020). We submit that ideology has the credentials to be taken as a criterion to define organizational forms, which in our scheme is at the most inclusive Level 4.

Exhaustive, universally accepted classifications of ideologies do not exist. However, one may trace a distinction between broadly defined categories such as ethical, political, religious, national, and corporate ideologies, and more specific instances such as gender, diversity, racial, market, educational, language, and even medical and nutrition ideologies. While the former constitute a rather stable set of classifications, the latter is more open to additions.

Quite often, ideology is a matter where top management is the initiator. Its hierarchical, top-down, homogenizing nature (Goll and Zeitz, 1991) suggests us to hypothesize that T-patterns^4^ may change abruptly when a new top management steers the organization toward a new vision and a new strategy.

## Emergence and diffusion

In this section, we reconstruct—whenever possible—the historical origin of the aforementioned organizational forms, highlighting their diffusion processes. More specifically, whenever possible, we shall attempt to reconstruct the structure of information flows that were at work.

In general, novel organizations are created all the time, whereas the emergence of a novel form is quite a rare event. New organizational forms appear relatively often at Level 1, in practice with every new production, marketing, or organizational technology. By contrast, the structural forms we listed at Level 2 are quite stable, although one may conceive of sub-forms that are conceived to fit specific niches. The institutional forms of Level 3 are even more stable, whereas the stability of the ideologically-defined forms of Level 4 depends on their ability to encompass a variety of phenomena.

Henceforth, the emergence and diffusion of novel organizational forms at the four generality levels will be discussed in the same sequence as in the previous section.

### Patterns of behavior

The emergence of a novel organizational form in terms of a novel behavior pattern is probably more common than organizational forms at a higher generality level. For businesses, it happens any time a novel technology generates a new industry with its own peculiar pattern of interactions between competing firms, their customers, suppliers, and other stakeholders.

The generation of novel behavior patterns involves several actors, including entrepreneurs as well as established firms and public agencies, quite often scientists or reformers who build up a community of practice where ideas are exchanged, tacit knowledge is made explicit, and technologies are developed and refined (Garud and Van de Ven, 1989; Romanelli, 1991; Fiol and Romanelli, 2012). BioTech is a particularly interesting point in case, with cross-contamination between molecular biologists and clinical physicians at the University of California, San Francisco, in mid-1970s acting as a fertile ground for the first companies that would exploit recombinant-deoxyribonucleic acid (DNA) technology (Jong, 2006). Scientific and technological breakthroughs were available, basic intuitions were being made explicit and published in scientific journals, and even managerial techniques were being discussed (Fairtlough, 1994). This is not a detail, because biotech is not based on hierarchically managed research projects such as those that the big pharmaceutical companies were used to manage, but rather thrives by allowing researchers a degree of freedom in proposing projects (James, 2000). Thus, it is really a novel organizational form based on a novel behavior pattern. From our point of view, one interesting aspect is that in spite of all the difficulties in understanding and reproducing it, within a couple of decades several agencies around the world had re-created similar companies and cultural environments (Niosi et al., 2013). This could happen because all the relevant knowledge was quickly codified, published, and even advertised by specialized consulting firms.

In the above example, information diffused rather quickly from its origin to the rest of the world. However, what is normal in contemporary societies may not have always been the case. Consider the following counter-example from the late Middle Age.

*Preci*, a tiny small village on the Italian Apennines, had developed what was possibly the most advanced surgical knowledge in Europe between the 13th and the 17th century. Their most distinctive secret was that by passing tools on a flame before operating, patients were less likely to die (Davidson, 2016). The nearby village *Cerreto* tried to imitate, but they either could not steal the secret or did not understand its importance (Timio, 2002). The word *charlatans* supposedly derive from crossing the name of its inhabitants, the *Cerretani*, with the onomatopoeic *ciarlare*, a dialect entry for *to chat* (Treccani, 2022).

Albeit a curiosity, this anecdote tells us that the structure of information flows might have been quite different in past ages. It tells us about a world where valuable knowledge was effectively kept secret, local, unexploitable by other actors. If it ever diffused, it did only through local interactions, slowly and imperfectly.

### Structure

At the level of organizational forms defined by their structure, we find four broad instances: The functional structure, the multidivisional structure, the matrix, and the adhocracy, or network organization. To our knowledge, no information is available regarding the origin and diffusion of the functional structure, which is perhaps too obvious for its invention to have been recorded. By contrast, we know that the multidivisional structure was invented in two US companies at roughly the same time, in the 1920s, in the two flavors with which this structure is still employed today.

The first company is *DuPont*, a producer of explosives that had made huge profits during World War I but had to reconvert its production once the conflict was over. With huge financial resources available, they diversified into chemicals ranging from colors to artificial fabrics and celluloid. To their surprise, they ended up in the red numbers because their single procurement, production, and sales departments had a hard time at delivering so diverse products on time. Aggregation had been supposed to provide scale economies, but timely delivery was much more important in the consumer goods markets they had just entered. Overcoming immense internal resistance and through a tortuous path of partial experimentations they finally arrived at creating separate divisions for explosives, colors, artificial fabrics, and other products. Historically, this is the first instance of a divisional structure defined by classes of products (Chandler, 1962).

The second company is *Sears & Roebuck*, which used to sell durable goods to rural America shipping them by railway. The Ford Model T changed forever the rules of the game, making it possible for peasants to reach big cities in order to procure the goods they would not find in their village. *Sears* reacted by duplicating its operations in five big stores at the periphery of five big cities, where goods were on sight on scaffolds along paths where customers could freely walk. Since each of these five stores had its own procurement and sales functions, they had invented the multidivisional structure based on geographical areas (Emmet and Jeuck, 1950).

The diffusion of the M-Form was very slow in the 1930s but took off after World War II. The number of firms adopting it increased linearly in the 1950s and 60s to reach saturation in the late 1970s, when nearly all large and diversified companies had adopted it (Teece, 1980). The speed of diffusion reflects different information channels, in the 1930s and 1940s the multidivisional structure used to spread by imitation, whereas since the end of World War II, it was being taught in business schools.

The matrix structure was theorized by management consultants in the 1970s out of the organizational structure of engineering contractors, which typically assign to each new project a team composed of personnel drawn from different functions. Thus, each team member has two bosses, the functional director and the project director. The matrix structure makes this arrangement permanent with the purpose of combining expertise located in different areas of the organization; however, its decision-making processes are typically longer and complex.

The matrix was the management fad of the 1970s. It was applied to a number of organizations for which it had not been appropriate, leading to its dismissal in the 1980s. However, since the 2000s it is enjoying a limited but rising popularity, once it has been understood that it is the proper structure when the value added by combining dispersed information is worth the necessarily more cumbersome decision process (Galbraith, 1971, 2009).

The conceptualization and diffusion of the matrix structure is remarkable in that it has been in the hands of a few global consulting firms. Its dismissal in the 1980s occurred spontaneously, in a wave just as emotional as the one that had brought it to the front. By contrast, its gradual resurrection since the 2000s has been largely managed by academics.

The adhocracy, or network organization, appeared in isolated cases in the 1950s and 1980s but took momentum with the new millennium. It is still poorly conceptualized and little understood, with consultants making use of a series of ever-changing buzzwords that are supporting a never-ending series of management fads (Cabri and Fioretti, 2022). In information-structural terms, it is a series of centrally-managed waves that trigger mass adoptions and mass dismissals without any deep understanding of the conditions and operating principles of this new organizational form.

### Institutional arrangements

The machine bureaucracy, the most common and most representative organizational form of modern societies, originated with the Industrial Revolution, in England, in the late XVIII century. Its distinctive emphasis on regulations and norms, as well as clear rights and duties for all of its members, mirrors the ideals of the French Revolution that turned subjects into citizens.

The machine bureaucracy is the organizational form of capitalism, and capitalism is, first of all, a way of thinking. It implies postponing leisure in order to accumulate resources to be invested in some enterprise that will hopefully yield a return in the future, sometimes a distant future. It is based on hard work, well beyond what is needed to enjoy the pleasures of life, and therefore requires, and induces, a mentality change.

In the late XVIII century, even in England, the capitalistic mentality was uncommon. A few early capitalists had it, but the vast majority of the population did not.

The earliest capitalists operated in the textile industry. Initially, they delegated production to contracted craftsmen. This putting-out system was subsequently abandoned in favor of factories where production was directly managed by the capitalists themselves.

According to Marglin (1974), factories had to be set up because craftsmen, with their pre-capitalist mentality, were unwilling to work long hours. They would not renounce leisure in order to earn more money in a world where, in any case, the variety of goods on offer was extremely limited. They would rather accept contracts insofar as they needed money to survive, devoting the rest of their time to inexpensive leisure. Their ideal was the idle noble, not the industrious entrepreneur.

However, nobles had changed a lot in the meantime. In the attempt to keep the pace with the rising capitalists, many of them had started to manage their land efficiently, which implied that all of it had to be put to productive usage. However, the traditional practice of leaving a portion of land unused and available to peasants did have a rationale. It was the peasants’ insurance against epidemics, wars, or any natural disaster that could plunge them into misery, for by leveraging on their extended families they could exploit that land and survive. Once the formerly unused land had been enclosed and properly managed, those who had fallen into misery had no choice but moved into cities where they would accept any job available. The urban proletariat was born, and it was essential for capitalism to make its first steps.

According to Marglin (1974), the first factories were built out of this combination of craftsmen unwilling to work as much as requested, land enclosures, and the availability of an urban proletariat. Later on, technical innovations made the factory also more efficient with respect to the putting-out system.

This novel organization, the factory, needed a novel management system because, for the first time in human history, time had become a scarce resource. There was simply no time to check all the details of what subordinates were doing.

Management by exception was invented in these factories, painstakingly, slowly, along more than a century out of continuous comparison between predictions and actual values (Pollard, 1965; Miles and Snow, 1994). Formalize everything, standardize everything, expect goals, and focus only on the exceptions were the means by which many workers could be controlled at a time. The machine bureaucracy, with its rules and norms, was invented in order to escape from the moving sands of micromanagement.

There was little or no awareness of this process. Practitioners invented or refined accounting techniques that slowly diffused from firm to firm, at a time when management itself was not a codified discipline but rather a bundle of how-to that did not command the respect enjoyed by classical culture (Pollard, 1965). It was, for about a 100 years, purely decentralized information diffusion, slowed down by inertia though by no means hampered by legal or institutional arrangement. Management by exception reached academic dignity only at the beginning of the XX century (Fayol, 1916), after it had been spontaneously adopted by all industrial enterprises.

Far less is known about the historical development of the professional bureaucracy. Medieval guilds have been an outstanding and pervasive instance, with the guilds regulating and codifying all the details of craft production. Precisely because of these regulations, technological innovation was nearly impossible in the Middle Age. Innovators would incur in harsh punishments, from prohibition to work up to ostracism and death.

It was not as absurd as it may appear at first sight. In a globally stationary economy, one innovation made by one craftsman would not have made the pie grow for everybody. Rather, it would have simply shifted resources from the non-innovators to the one innovator. Thus, it was natural for the vast majority of non-innovators to oppose any such move.

However, exceptions did exist. For instance, Benedectine monasteries were not subject to the guilds and therefore they could innovate agricultural techniques, as well as food processing machines (Kieser, 1989). In structural terms, Benedectine monasteries constituted a network of sources of information that let it diffuse into the rest of society.

Modern professional bureaucracies are not as extreme. Max Planck remarked that Science proceeds only after an old generation of scientists has disappeared (Planck, 1950), but in spite of all difficulties scientific revolutions do take place in the end. In this environment, innovative and open-minded journals and researchers’ communities may have a similar role as the Benedectine monasteries of the Middle Age.

Identifying the origin of professional bureaucracies may prove to be an impossible task, but evidence of the existence of crafts that standardized technologies and excluded non-members can be traced back to the neolithic age (Sterelny, 2012). Possibly, the professional bureaucracy simply originated with the first tools Man was able to make.

Even less is known about patronage-based organizations. It is known that it was the default organizational form of the kingdoms and empires that existed before the Industrial Revolution, though such a claim should be tempered by the observation that some degree of standardization did take place at key milestones of human history such as Hammurabi’s written Code, or Roman Law.

It is known that the first large human settlements originated with the invention of hydraulic agriculture, first with the Sumers about 7,000 B.C.E. and then quite independently along the Nile, the Yellow River, and elsewhere. The possibility of irrigating fields during the dry season enabled those populations to build permanent settlements, cities, and empires whose administrations have been the first large organizations in human history.

We also know that in primitive or archaic societies reciprocity was the fabric that kept humans bound to one another. Exchanging gifts was the ritual that ensured that community members entertained good relationships with one another (Sahlins, 1972).

By combining the two above pieces of knowledge, it has been speculated that patronage originated from gift exchange once the command over valuable resources had created hierarchies that deprived it of its original equality. Exchange was still making for good relations, but the items being exchanged had changed a lot. No longer goods of equal value, but favors in exchange for dependence (Hooper et al., 2018).

The structure of information flows implied by the above reconstruction is quite remarkable, for it is made of multiple independent origins—the Tigris-Euphrates, the Nile, the Yellow River, etc.—that emanated one and the same new organizational form, which subsequently diffused without any sort of information centralization. Obviously, multiple origin was made possible by the supposed ubiquity of gift exchange.

### Ideology

In organization studies, terms like ideology, strategy, and tactics differ in grade of detail, closeness to practice, and long- vs. short-term orientation, yet they all denote some form of cognitive framework for taking action (Smithey, 2009; Mackay and Zundel, 2017). Their origin can be ascribed to the innate drive of the human mind to seek coherence, constructing networks of causal linkages that provide orientation in spite of a necessarily uncertain future, sometimes even at the cost of distorting reality to some extent (March and Olsen, 1976; Weick, 1979, 1995; Lane and Maxfield, 2005). As such, ideologies are likely to have been with mankind since the beginnings.

However, the structure of their diffusion might have changed with time. In particular, there exists a very recent trend toward short-lived, divisive ideologies that are almost devoid of content in comparison to those prevailing in the XX century, and sometimes even based on fake news (Freeden, 2019). Their diffusion is often different from the propagation from one single center that characterized the ideologies of the past. Propagation most often occurs through social communication media, where it stems from a small number of hubs that are linked to one another by channels that may use any means, including personal acquaintance (Zhu et al., 2016). Moreover, a similar structure appears to operate within organizations, where a typically small number of members becomes engaged with information hubs (Majchrzak et al., 2013).

## Discussion

With this paper we summarized the available knowledge about organizational forms, arranging the existing definitions in a hierarchy of four levels nested in one another. In spite of lack of data, we speculated on possible features of their T-patterns and reconstructed qualitative features of their diffusion. In particular, we identified the following diffusion patterns:

1. Diffusion from one or a few sources relying on local connections, as it has largely been the case for the machine bureaucracy. This diffusion mode is, quite obviously, very slow.
2. Diffusion from one source with global connections, as it has been the case, for instance, for the diffusion of the ideologically-defined Communist States out of the template provided by the Soviet Union. This diffusion mode is likely to be faster than case (1).
3. Diffusion from a few sources with global connections and closely linked to one another, as it has been the case, for instance, of the diffusion of the matrix structure operated by a few global consultants. Assuming some degree of coordination between the original sources, this diffusion mode is likely to be faster than case (2).
4. Diffusion out of several sources that independently arrived at the same arrangement, as it may have been the case for patronage-based organizations arising out of spontaneous reciprocity once a social hierarchy had been put in place. Differently from case (3), in this case the sources are assumed not to coordinate with one another, hence speed of diffusion may be lower than in case (3).

Sometimes, these three modes are exploited in sequence. For instance, the organizational form of biotech firms was initially conceived by local actors and started diffusing spontaneously in the San Francisco Bay area, but it was eventually picked up by global consultants. In this case, the diffusion mode switched from (1) to (3). Likewise, the diffusion of the multidivisional form initially took place by imitation of two firms that had independently invented it, to be subsequently picked up by global consultants. In this case, the diffusion mode switched from (4) to (3). We may also speculate that many instances of mode (3) diffusion actually started as (2), with one single hub being subsequently imitated by other hubs. Apparently, in quite many cases (3) may have been an attractor toward which other diffusion modes converged over time.

Quite obviously, our conceptual scheme has limitations. We already mentioned the problem of organizational hybrids, which is particularly serious for adhocracies/network organizations but to some extent concerns all organizational forms. Multidivisional structures, for instance, are generally hybridized with functional structures because at least one of their functions—most often, finance—generally remains centralized. Likewise, hospitals and universities are no longer the pure professional bureaucracies they used to be, for they are hybridizing themselves with machine bureaucracies and network organizations (Lega and DePietro, 2005). Our scheme can help accommodate hybridizations along the vertical dimension—from Level (1) to Level (4)—but horizontal hybrids remain a nuisance for the theory, just like attrition for the laws of physics.

A similar difficulty arises with organizations whose portions belong to different organizational forms. This is for instance the case of large machine bureaucracies, whose top hierarchical level is generally based on patronage, as well as large multidivisional companies whose divisions may pertain to different forms in terms of their behavior patterns. It is inevitable that, in such cases, local or partial belongingness must be considered.

Quite similarly, in many instances, a degree of belongingness to ideal-typical organizational forms should be introduced. There is clearly a degree to which an ideology is believed, and real machine bureaucracies conform to the Weberian ideal type only to some degree. Note, however, that to the extent that a lower-than-100% belongingness to an organizational form obtains because of partial belongingness to a different organizational form, we are back to the problem of organizational hybrids.

On the whole, we are very cautious concerning the validity of our hypotheses concerning T-patterns, particularly those of the highest levels. By contrast, we suspect that there exists a real drive toward diffusing information by coordinating a few sources, each of which is endowed with global connections. From Benedectine monasteries to social media, this communication structure possibly turned out to be most effective.

## Author contributions

MN contributed to knowledge from sociology. GF contributed to knowledge from management studies. The paper evolved in several rounds of discussion in which the authors tried to arrive at a unified framework that included knowledge from both domains. All authors contributed to the article and approved the submitted version.

## Conflict of interest

The authors declare that the research was conducted in the absence of any commercial or financial relationships that could be construed as a potential conflict of interest.

## Publisher’s note

All claims expressed in this article are solely those of the authors and do not necessarily represent those of their affiliated organizations, or those of the publisher, the editors and the reviewers. Any product that may be evaluated in this article, or claim that may be made by its manufacturer, is not guaranteed or endorsed by the publisher.
